# Interleukin-4-Mediated 15-Lipoxygenase-1 Trans-Activation Requires UTX Recruitment and H3K27me3 Demethylation at the Promoter in A549 Cells

**DOI:** 10.1371/journal.pone.0085085

**Published:** 2014-01-20

**Authors:** Hongya Han, Dawei Xu, Cheng Liu, Hans-Erik Claesson, Magnus Björkholm, Jan Sjöberg

**Affiliations:** 1 Department of Medicine, Division of Hematology, Karolinska University Hospital Solna and Karolinska Institutet, Stockholm, Sweden; 2 Department of Urology, Qilu Hospital, Shandong University, Jinan, PR China; Università degli Studi di Milano, Italy

## Abstract

Arachidonate 15-lipoxygenase-1 (ALOX15) oxygenates polyunsaturated fatty acids and bio-membranes, generating multiple lipid signalling mediators involved in inflammation. Several lines of evidence indicate that ALOX15 activation in the respiratory tract contributes to asthma progression. Recent experimental data reveals that histone modification at the promoter plays a critical role in ALOX15 gene transcription. In the present study, we examined the status of histone H3 trimethyl-lysine 27 (H3K27me3) at the ALOX15 promoter by chromatin immunoprecipitation assay in human lung epithelial carcinoma A549 cells incubated with or without interleukin (IL)-4. We identified demethylation of H3K27me3 at the ALOX15 promoter after IL-4 treatment. Furthermore, we found that the H3K27me2/3-specific demethylase, ubiquitously transcribed tetratricopeptide repeat, X chromosome (UTX), mediates the H3K27me3 demethylation during ALOX15 transcriptional activation. When UTX expression was knocked down using siRNA, IL-4-mediated H3K27me3 demethylation and ALOX15 induction were significantly attenuated. The critical role of UTX in ALOX15 expression was confirmed in human monocytes and the Hodgkin lymphoma (HL) cell line L1236, but was in these cells not related to H3K27me3-demethylase activity. These results demonstrate that UTX is implicated in IL-4 mediated transcriptional activation of the ALOX15 gene.

## Introduction

Arachidonate 15-lipoxygenase (ALOX15) is an enzyme that oxygenates polyunsaturated fatty acids and bio-membranes. In healthy subjects, this enzyme is predominantly expressed in airway epithelial cells, eosinophils, alveolar macrophages, dendritic cells and reticulocytes [1]. In human peripheral monocytes, the transcription and expression of ALOX15 is induced upon interleukin (IL)-4 and IL-13 stimulation [2], [3]. Induction of ALOX15 by IL-4 was also observed in lung epithelial carcinoma A549 cells [4]. In the ALOX15 cascade, multiple lipid metabolites are generated. These small molecules are potent signalling mediators, involved in various inflammatory diseases and cancers [1], [5], [6]. In several studies, ALOX15 has been suggested to contribute to asthma pathogenesis and increased expression of epithelial ALOX15 associates with increased disease severity [1], [7]–[9]. In certain human cancers, such as colon, oesophageal, and pancreatic cancer, the expression of this enzyme is suppressed and has been suggested as a tumour suppressor [10]–[14]. In contrast, ALOX15 is highly expressed in the tumour cells of Hodgkin lymphoma (HL), the so-called Hodgkin Reed-Sternberg (H-RS) cells [15], and prostate cancer cells [16].

The expression of ALOX15 is strictly regulated in normal and cancerous human cells. The T-helper type 2 cytokines IL-4 or IL-13activate signal transducer and activator of transcription-6 (STAT-6), which subsequently binds to the promoter, and drives ALOX15 mRNA transcription [2], [4], [17]–[19]. Recently, experimental evidence revealed that histone modification plays an important role in ALOX15 transcriptional regulation. Histone H3 and histone H4 acetylation mediated by histone acetyltransferase lysine (K)-acetyltransferase 3B (KAT3B), and histone H3 dimethyl-lysine 9 (H3K9me2) demethylation catalysed by lysine (K)-specific demethylase 3A (KDM3A) transcriptionally activate ALOX15 in colon cancer cells [20]. In a previous study, we found that the histone methyltransferase SET and MYND domain-containing protein 3 (SMYD3) is expressed in the HL cell line L1236 that is ALOX15 positive, while in the ALOX15 negative HL cell line L428, expression of SMYD3 protein is not detectable, although the histone demethylase SMCX is expressed. SMYD3 and SMCX regulate ALOX15 gene expression by controlling histone H3 at lysine-4 (H3K4) methylation status in HL-derived cell lines[21].

Histone methylation is a critical epigenetic mechanism, which regulates embryonic development[22], immune responses[23], [24], and cancer genesis[25], [26]. Histone methylation plays a pivotal role in the maintenance of both active and suppressed states of gene expression depending on the sites of methylation [27]. The methylation of histone H3 at lysine-27 (H3K27) is implicated in repression of gene transcription[28]. Jumonji domain containing 3 (Jmjd3) and ubiquitously transcribed tetratricopeptide repeat gene, X chromosome (UTX), both members of the JmjC protein family, are key specific demethylases of H3K27me2/3[29]. Jmjd3 is induced by lipopolysaccharides in mouse macrophages to regulate macrophage activation and implicated in generation of the IL-4-induced alternatively activated macrophage M2 phenotype [27], [30]. UTX was recently found in a complex with MLL2 that mediates transcriptional activation of the HOX promoters [29], [30].

Among the ALOX15 gene regulatory mechanisms, IL-4-induced ALOX15 expression through the STAT-6 cascade is the most studied signal transduction pathway [31]. The HDAC inhibitor suberoylanilide hydroxamic acid (SAHA) supresses STAT-6 expression [32], but stimulates ALOX15 [33], suggesting the importance of histone modification that might act as an additional or alternative pathway in ALOX15 transcriptional activation. However, the presence and importance of IL-4-mediated histone modification in ALOX15 induction is unknown. In the present study, we examined histone modification upon IL-4 stimulation in A549 cells. In particular, the status of H3K27me3 at the ALOX15 promoter was assessed by chromatin immunoprecipitation (ChIP) assay. We identified a novel histone modification at the ALOX15 promoter that activates ALOX15 transcription via UTX recruitment and H3K27me3 demethylation in A549 cells. The implication of UTX in ALOX15 regulation was also studied in human monocytes and cultured HL cells.

## Materials and Methods

### Cell lines and culture conditions

The lung epithelial carcinoma cell line A549 (Leibniz-Institute DSMZ-German Collection of Microorganisms and Cell Cultures, Brunswick, Germany), and the human HL cell line L1236 (kind gift from Prof. Volker Diehl, Cologne, Germany) were cultured at 37°C in RPMI 1640 containing 10% fetal calf serum, 100 units/mL penicillin, and 2 mM L-glutamine (Invitrogen, CA, USA). HEK293FT cells (kind gift from Dr. Shanzheng Yang, Department of Medical Biochemistry and Biophysics, Karolinska Institutet, Stockholm, Sweden) was cultured in D-MEM (high glucose) containing 10% fetal bovine serum (FBS), 0.1 mM MEM Non-Essential Amino Acids (NEAA), 6 mM L-glutamine, 1 mM MEM Sodium Pyruvate, and 500 µg/ml Geneticin (Invitrogen, CA, USA).

### Human monocytes isolation and culturing

Peripheral blood mononuclear cells (PBMCs) were isolated from anonymised buffycoats from healthy blood donors at the Karolinska University Hospital by Ficoll-separating solution density gradient centrifugation. Monocytes were isolated by negative selection; a Monocyte Isolation Kit II with an LS column was used according to the manufacturers' instructions (Miltenyi Biotec Inc. Auburn, CA, USA). The isolated monocytes were cultured in RPMI including 5% AB serum and supplied with IL-4 as indicated.

### RNA extraction and reverse transcription and quantitative real-time PCR (qRT-PCR)

RNA was isolated using TRIzol (Invitrogen, CA, USA) according to the manufacturer's instructions. Total RNA was reverse-transcribed to yield cDNA using random primers (N6) and M-MLV reverse transcriptase (Invitrogen, CA, USA) as previously described [34]. qRT-PCR analysis was performed by using SYBR green (Applied Biosystems, CA, USA) under 7900 Real-Time PCR System (Applied Biosystems, CA, USA). qRT-PCR for Glyceraldehyde-3-phosphate dehydrogenase (GAPDH) expression was run in parallel as a control for loading and PCR efficiency. The primers for the qRT-PCR are listed in table? 1.

**Table 1:** Primer sequences.

Primers	Sequences(5′-3′)	
ALOX15 (qRT- PCR)	Forward	ACTGAAATCGGGCTGCAAGGG
	Reverse	GGGTGATGGGGGCTGAAATAA
UTX (qRT- PCR)	Forward	TAC AAA TCC GAA CAA CCC
	Reverse	TGA GGA GGC CTG GTA CTG T
GAPDH (qRT- PCR)	Forward	CCG GGA AAC TGT GGC GTG ATG G
	Reverse	AGG TGG AGG AGT GGG TGT CGC TGT T
ChIP Primer-Pair 1 (Region ???)	Forward	GCATGCTACAATGTGCAGAAA
	Reverse	CTCCACTCCCAGCAATCACA
ChIP Primer-Pair 2 (Region ???)	Forward	CTGGGGAACACATGGCTCCAG
	Reverse	CCAAATGTTTCTGGCATTCAA
ChIP Primer-Pair 3 (Region ???)	Forward	CCTCGCCCACTTCACCCTCTC
	Reverse	AGCCTCACAAGTTGGATTGCA
ChIP Primer-Pair 4 (Region ???)	Forward	CCCACCTCGCCTGCCTGCTGTA
	Reverse	TGGCAGGTCTCCAATCAACTC

### Immunoblotting

Total cellular protein was extracted with RIPA (Santa Cruz Biotechnology, Santa Cruz, CA, USA). Thirty microgram of the protein were resolved by 4–20% Mini-PROTRAN®TGX™ Precast Gels and transferred to a PVDF membrane by Trans-Blot® Turbo™ Mini PVDF Transfer Pack (Bio-Rad Laboratories, CA, USA). The membrane was probed with ALOX15 rabbit peptide antiserum (batch 632) raised against the ALOX15 peptide CALDKEIEIRNAKLDMPYEY (antibodies made by Innovagen AB, Ideon, Lund, Sweden. The antibody did not cross-react with ALOX5 or ALOX12 (data not shown)), UTX (Bethyl Laboratories, Inc, Montgomery, TX, USA) or β-actin (Santa Cruz Biotechnology, Santa Cruz, CA, USA) followed by anti-rabbit or mouse horseradish peroxidase–conjugated IgG and developed with the enhanced chemiluminescent method (GE healthcare, UK).

### siRNA and overexpression vectors transfection

Chemically modified Stealth siRNA targeting UTX (GGUCUUGGUUUUGGUCUACUUCCAUU), STAT-6 (GGGAGAAGAUGUGUGAAACUCUGAA) and Stealth™ RNAi Negative Control were purchased from Invitrogen (Invitrogen Carlsbad, CA, USA). The UTX expression vector pCMV-HA-UTX was a kind gift from professor Kristian Helin, University of Copenhagen. siRNAs and vectors were transfected into A549 cells using Lipofectamin 2000 (Invitrogen, CA, USA) according the manufacturer's protocol.

### Lentivirus production and transduction

Monocytes and HL cell line L1236 cells were transduced with lentivectors containing UTX specific or scrambled short hairpin RNA. For monocyte transduction, the helper simian-immunodeficiency-virus-like-particle (SIV-VLP) was used according to a previously reported method[35]–[37], Briefly, the HEK293FT cells were plated at the concentration of 1.5×10^6^ cells/well in 6-wells plates, and incubated at 37°C, 5% CO2 overnight. To produce lentivirus, the scrambled vector pLKO.1 or pLKO.1-shUTX (Clone ID: TRCN0000107760)(Thermo Fisher Scientific Inc., MA, USA) were transfected together with packaging system, psPAX2 and pMD2.G (Addgene, MA, USA) into HEK293FT cells. SIP-VLP was produced by transfecting pSIV3+, pMD2.G into HEK293FT cells. The lentivirus and SIV-VLP containing supernatant was collected and filtered. Simultaneously, human peripheral monocytes were isolated according to the monocyte isolation protocol as stated above. Purified monocytes (purity over 90%) were then seeded into 6-wells plates (3×10^6^ cells in 3 ml monocyte culture medium containing 50 ng/ml IL-4) and the lentivirus together with helper SIV-VLP were added to the culture. To transduce L1236 cells, the lentivirus containing supernatant was concentrated by using PEG-it™ Virus Precipitation Solution (System Biosciences, CA, USA) according to the manufacturer's protocol. L1236 cells were then transfected at MOI 8.

### ChIP assay

ChIP was performed using ChIP Assay Kit (Millipore, MA, USA) according to the manufacturer's protocol. Briefly, cells were cross-linked by incubating with 1% (v/v) formaldehyde-containing medium for 10 min at 37°C and then sonicated to obtain soluble chromatin with DNA fragments between 400 and 1,000 base pairs. Antibodies against H3K27me3 (GAH-9205, Qiagen GmbH, Hilden, Germany) and UTX (Bethyl Laboratories, Inc, Montgomery, TX, USA) were used to precipitate DNA fragments bound by their corresponding elements. As an additional control for nonspecific binding, the samples were also immunoprecipitated with non-immune rabbit IgG (GAH-9205, Qiagen GmbH, Hilden, Germany). The protein-DNA complex was precipitated with protein A sepharose beads, eluted, and reverse cross-linked. Following treatment with Protease K, the DNA was purified with QIAquick PCR Purification Kit (Qiagen GmbH, Hilden, Germany). The recovered DNA was resuspended in H_2_O and used for PCR amplification with primer sets (see table? 1). GAPDH primers (GAH-9205, Qiagen GmbH, Hilden, Germany) were used as a negative control.

### Statistical analysis

Student's t test was used for determination of statistical significance. All statistical analyses were performed using GraphPad Prism 5.0 software.

## Results and Discussion

IL-4 triggers mRNA transcription and protein expression of ALOX15 in A549 cells. However, while binding of the cytokine to its cognate surface receptor is required for gene expression, it is not sufficient in all cells expressing functional IL-4 receptors [4], suggesting more complex mechanisms involved in the induction process. In order to address the specific mechanisms, A549 cells were established as a model for inducible ALOX15 gene expression, which was confirmed at the mRNA and protein levels. The results showed that ALOX15 is induced by IL-4 in a dose- and time-dependent manner (Fig.? 1).

**Figure 1:**
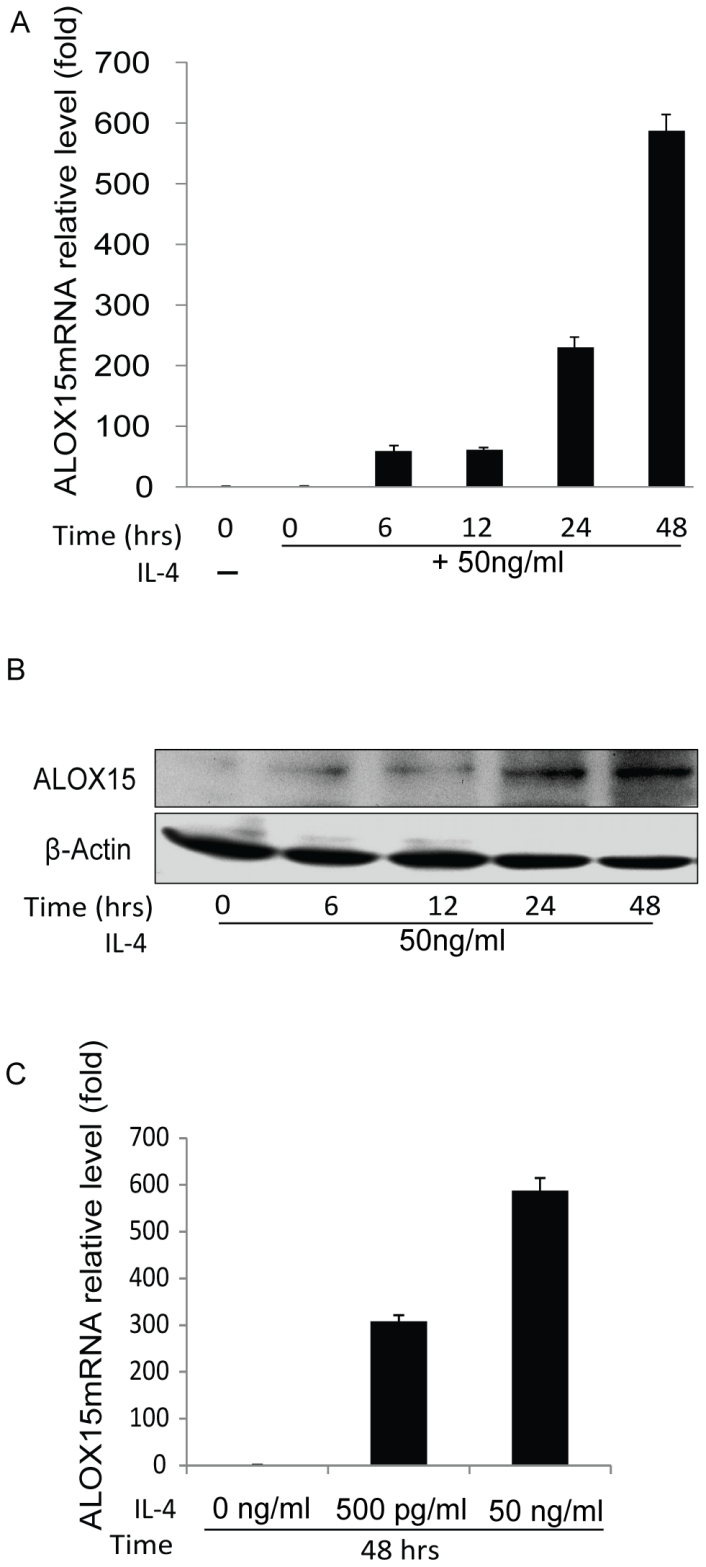
IL-4 induces ALOX15 expression in A549 cells. A549 cells were cultured in RPMI 1640 medium containing 50/mL IL-4 for indicated time points. (A) Total RNA was purified and ALOX15 and UTX mRNA was measured by qRT-PCR; (B) and the protein level of ALOX15 was measured by Western blot; (C) the dose effects of IL-4 on ALOX15 induction was determined by qRT-PCR. The western blot results represent two independent experiments, and the error bars represent standard error mean of three independent qRT-PCR experiments.

**Figure 2:**
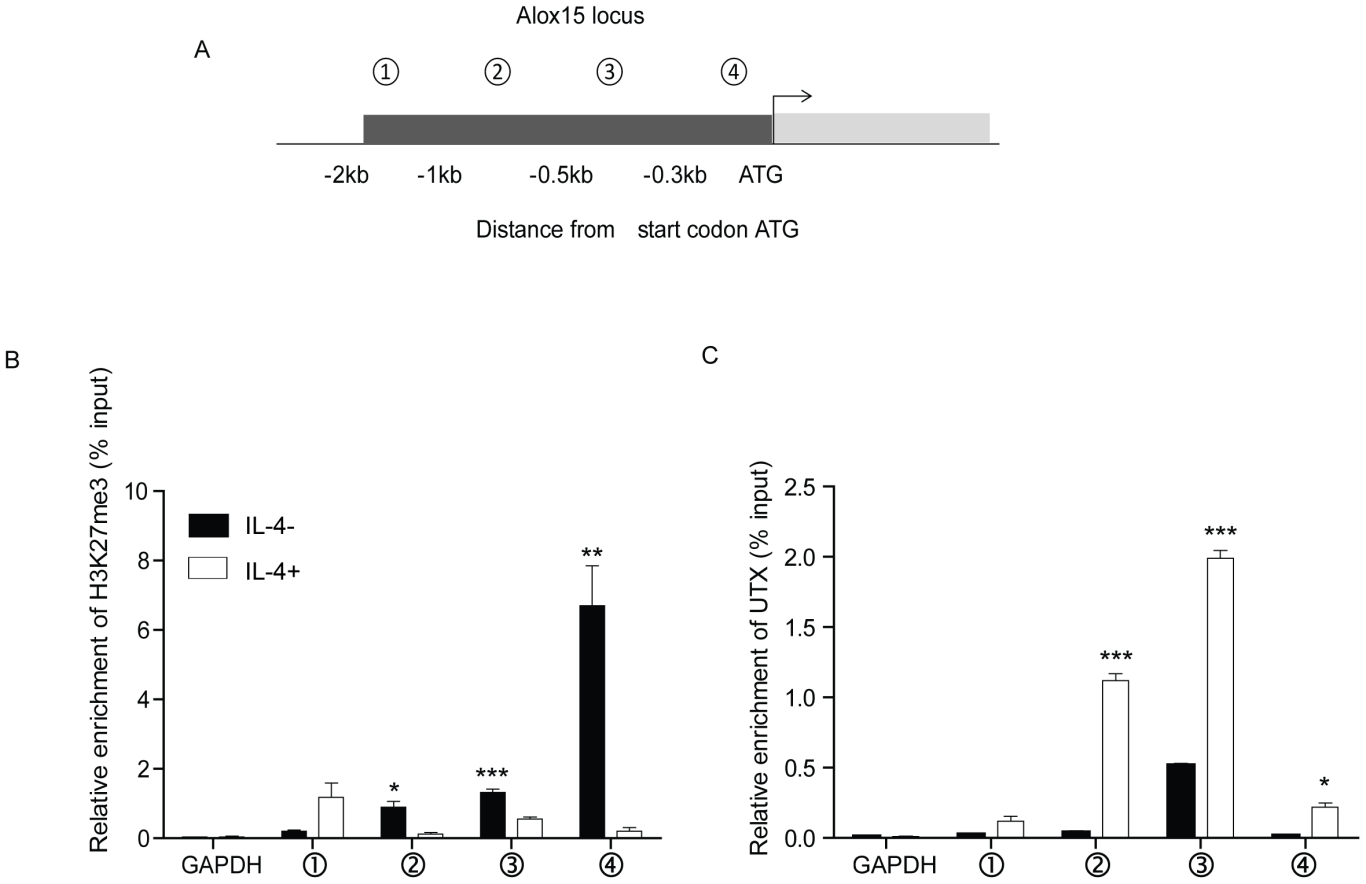
IL-4 mediates recruitment of UTX and histone modification at the ALOX15 promoter. (A) Schematic presentation of the ALOX15 promoter and locations (relative to ATG) of PCR primers used for ChIP assay; (B) Anti-H3K27me3 ChIP assay covering the indicated ALOX15 promoter regions was performed in A549 cells after IL-4 stimulation; (C) Anti-UTX ChIP assay was performed on the indicated ALOX15 promoter regions in A549 cells after IL-4 stimulation. The GAPDH primers were used as a negative control. Bars represent mean value of ChIP signals normalized to 1% input. Error bars represent standard error mean of three independent experiments. *p<0.05; ** p<0.01; *** p<0.001.

Chromatin modification has been reported to be important for ALOX15 transcriptional activation in certain cells[20]. Recent studies have shown that the H3K27 demethylases Jmjd3 and UTX regulate target genes dynamically upon continuous stimulation by IL-4 or LPS [27], [29], [30]. However, whether IL-4 induces histone modification at the ALOX15 promoter is unknown. Previous studies suggested that the DNA-protein binding regions are located within 500bp upstream of the ATG start codon [18], [19]. Therefore, the H3K27 trimethylation level of the ALOX15 promoter upon IL-4 stimulation was tested. A549 cells were incubated with IL-4 for 48 hours followed by ChIP analysis and qRT-PCR by using four pairs of primers along the promoter of ALOX15 as indicated (Fig.? 2A). The ChIP results showed that the non-IL-4-treated A549 cells exhibit relative high levels of H3K27me3 in core DNA-protein binding regions of ALOX15 promoter, and that the IL-4 treatment substantially reduced the H3K27me3 level in these regions. The specificity of the assay was verified by the absence of specific sequence amplifications when H3K27me3 specific antibody was replaced by pre-immune IgG in the procedure, or when primers for the unrelated GAPDH gene were applied in the PCR reaction (Fig.2B). These results indicate that IL-4 mediates H3K27me3 demethylation of the ALOX15 promoter in the DNA-protein binding regions in A549 cells.

Tri-methylation of H3K27 is a repressive marker of gene transcription [27], [28]. Methylated H3K27 is a target for Polycomb-Repressive Complexes (PRCs) that silence gene expression[28]. In untreated A549 cells, ALOX15 is not expressed. We hypothesized that occupancy of PRCs at the ALOX15 promoter might contribute to the silencing of the gene in these cells. To identify the mechanism/s by which IL-4 mediates H3K27me3 demethylation of the ALOX15 promoter, the expression of the two main H3K27me3 demethylases, UTX and Jmjd3 [29], were examined using qRT-PCR and Western-blot. While Jmjd3 was hardly detectable, a relatively high level of UTX was detected in untreated A549 cells, although IL-4 treatment did not increase the expression (data not shown). However, enrichment of UTX at the ALOX15 promoter was observed upon IL-4 stimulation by using ChIP assay. UTX was binding at the promoter region between -1000bp to -300bp (relative to start the codon) (Fig.2C), which covers one of the reported DNA-protein binding regions.[18].

**Figure 3:**
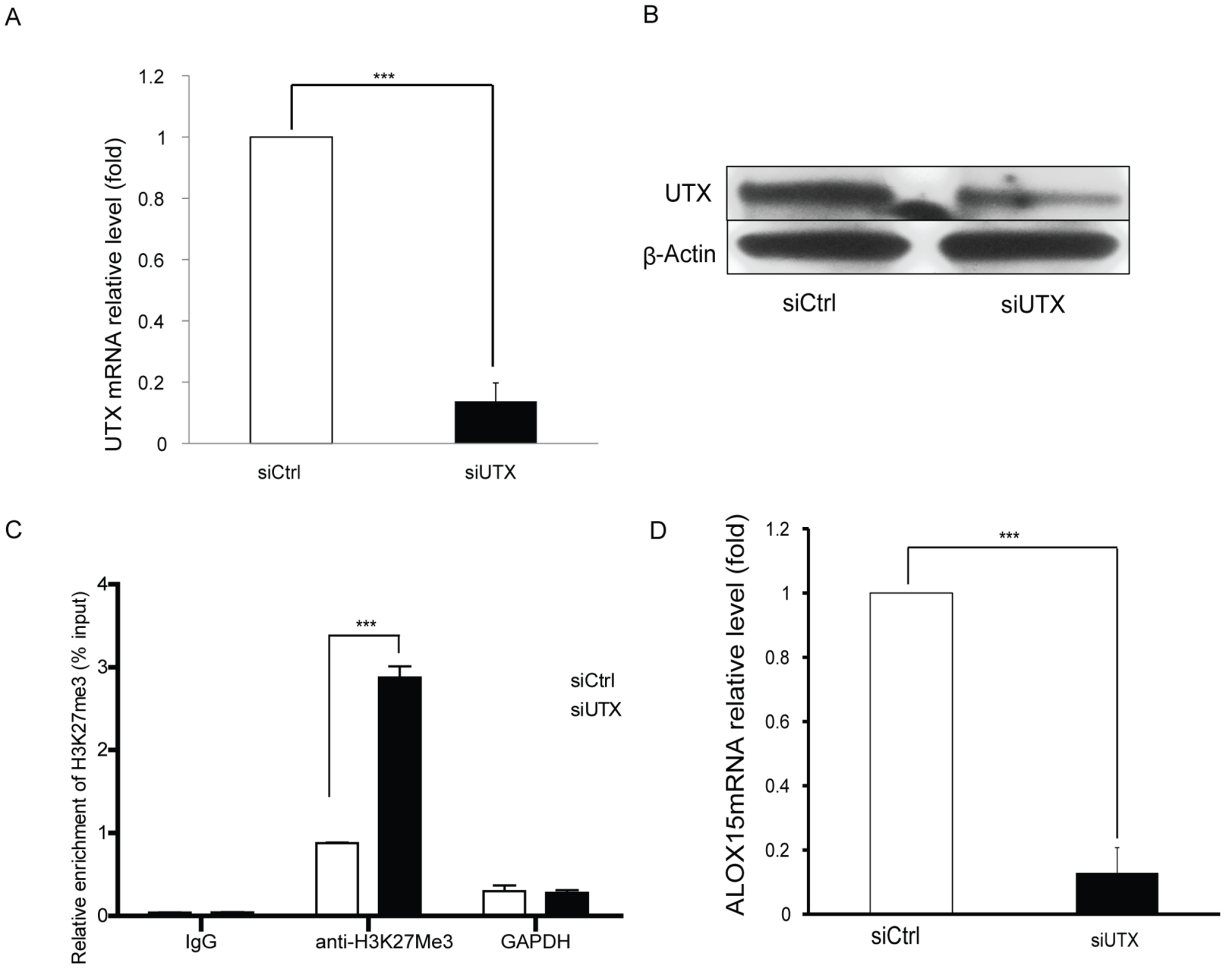
Lysine (K)-specific demethylase UTX is required for ALOX15 induction in A549 cells. UTX specific siRNA was transfected 72-4 treatment, and total RNA and protein were purified, followed by qRT-PCR and Western blot, the mRNA (A) and protein level (B) of UTX was measured upon UTX depletion followed by IL-4 stimulation. (C) The status of H3K27me3 at ALOX15 promoter region 3(see figure? 2) was verified upon UTX depletion followed by IL-4 stimulation; (D) the effect of UTX depletion on IL-4-induced ALOX15 expression was measured by qRT-PCR. All qRT-PCRs used GAPDH as loading control and the relative expression levels were calculated as the values relative to those of the calibrator samples (untreated sample). β-Actin was used as a loading control for all western blots. qRT-PCR data is shown as “fold induction” relative to that in control cells. Error bars represent standard error mean of three independent experiments. *p<0.05; ** p<0.01; *** p<0.001.

In order to verify the importance of UTX recruitment in ALOX15 induction upon IL-4 stimulation UTX specific siRNAs were applied prior to IL-4 treatment. Efficient suppression of UTX was confirmed by qRT-PCR and Western blot (Fig.? 3A and B). As UTX was mainly binding at one of the DNA-protein binding regions (−500bp to −300bp) we assessed the H3K27me3 status at this region upon UTX inhibition. Depletion of UTX significantly attenuated demethylation of H3K27me3 and reduced ALOX15 induction by IL-4 (Fig.3C? and D). This indicates that UTX is a key demethylase implicated in IL-4 mediated H3K27me3 demethylation of the ALOX15 promoter in A549 cells. Upon over-expression of UTX in A549 cells by transfecting a UTX expression vector, however, ALOX15 expression was not induced (data not shown), which indicates that global expression of UTX alone is insufficient to activate ALOX15 transcription. Taken together, these results suggest that IL-4 signaling mediates specific recruitment of UTX to the ALOX15 promoter, and subsequently demethylation of H3K27me3 and ALOX15 transcriptional activation.

In the classical IL-4 pathway, receptor binding eventually leads to translocation of STAT-6 to the nucleus and transcriptional activation of genes responsive to the cytokine. The present study demonstrates histone modification upon IL-4 stimulation and it is considered of great interest whether these two pathways interact with each other. The polycomb group (PcG) and trithorax group (TrxG) complexes exert opposing functions on the maintenance of the transcriptional status of target genes: PcG proteins are involved in chromatin-based gene silencing via the methylation of histone H3K27, while TrxG proteins counteract the silencing effects on chromatin to maintain gene activity through the induction of methylation at histone H3K4[23], [28], [38]. Interestingly, the H3K27me3 demethylase UTX was also found to be associated with the TrxG complex [30]. A recent study demonstrated that during Th2 cell differentiation, IL-4 initiates the IL-4/STAT-6 pathway, which triggers the activation of the TrxG complex, mediating displacement of the PcG complex and leading to the induction of GATA3 transcription [39]. In addition, inhibition of the histone methyltransferase SMYD3 leads to chromatin remodelling and reduced STAT-6 occupation at the ALOX15 promoter, and SMCX suppression enhances STAT-6 binding at the ALOX15 promoter[21]. Thus, a potential association between the IL-4-STAT-6 axis and histone modification might exist also during ALOX15 induction in A549 cells.

However, this seems not to be the case in colon cancer cells. In Caco-2 and SW480 cells, depsipeptide treatment induces ALOX15 expression by the recruitment of KDM3A and KAT3B to the ALOX15 promoter, demethylating H3K9me2 and acetylating histone H3 and H4. Pre-treatment with STAT-6 siRNA demonstrated that STAT-6 is not necessary for depsipeptide-induced ALOX15 expression in colon cancer cells[20].

In A549 cells, it was confirmed that STAT-6 is of importance for ALOX15 induction (Fig.S1 A, B and C). However, we noticed that STAT-6 did not influence UTX transcription in these cells (Fig.S1 D). Further studies are needed to evaluate the relationship between UTX recruitment and STAT-6 association at the ALOX15 promoter.

**Figure 4:**
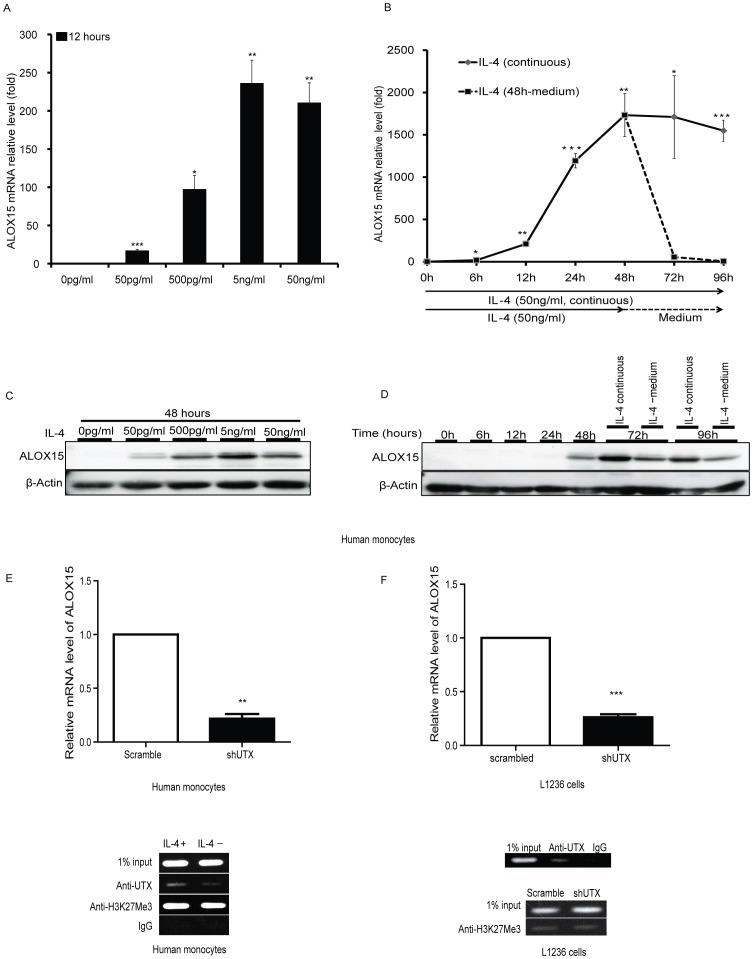
The expression of ALOX15 in human peripheral monocytes and HL-derived L1236 cells requires UTX. Human monocytes were treated as indicated. Quantitative RT-PCR analysis and Western blot were performed. (A) The dose response effect of IL-4 (12 hours) on ALOX15 mRNA induction; (B) the time course of ALOX15 transcription in response to IL-4 (50 ng/ml) stimulation; (C) the dose response effect of IL-4 (48 hours) on ALOX15 protein expression; (D) the time course of ALOX15 expression in response to IL-4 (50 ng/ml) stimulation. (E, upper-panel) Monocytes were transduced with UTX-specific or scrambled short hairpin RNA by means of a lentiviral vector, and simultaneously supplied with IL-4 (50 ng/ml) containing medium. The monocytes were harvested and quantitative RT-PCR analyses performed after five days culture. (E, lower panel) Monocytes were stimulated with IL-4 (50 ng/ml) for 24 hours and thereafter ChIP assay was carried out by using anti-H3K27me3 and anti-UTX antibodies, and quantitative PCR was performed using the primers covering the ALOX15 promoter region 3 (see figure? 2). (F, upper-panel) L1236 cells were transduced with either UTX-specific or scrambled short hairpin RNA by using lentiviral vector, and cells were harvest, total RNA were purified and quantitative RT-PCR analyses were performed after 7-days since transduction. (F, lower panel) L1236 cells were analyzed by ChIP assay using anti-H3K27me3 and anti-UTX antibodies, and followed by PCR using the primers covering the ALOX15 promoter region 3 (see Figure? 2). Rabbit IgG was used in ChIP as a negative control. Quantitative RT-PCR data is shown as “fold induction” relative to that in controls. Error bars represent standard error mean of three independent experiments. The Western blot and ChIP assay results represent three independent experiments. *p<0.05; ** p<0.01; *** p<0.001.

ALOX15 is one of the top IL-4-induced genes in human peripheral monocytes [40] and occurred in a dose- and time-dependent fashion (Fig.4A-D). Compared to A549 cells, the effect of IL-4 on ALOX15 induction was more potent in monocytes. Stimulation of monocytes with 50 ng/ml of IL-4 for 12 hours induced an approximately 210-fold upregulation of ALOX15 mRNA levels (Fig.4A) compared to only a 50-fold upregulation in A549 cells under the same conditions (see fig.1). In time course studies, a significant ALOX15 mRNA induction was observed as early as after 6 hours, and the logarithmic phase was reached by 12 hours (Fig.4B). Detectable protein levels of ALOX15 were observed after 24 hours IL-4 treatment (Fig.4C,? D). Interestingly, IL-4 withdrawal (replacement with IL-4 free medium after 48 hours culture) dramatically decreased the mRNA level of ALOX15 in 24 hours. However, the ALOX15 protein was relatively stable under these conditions, indicating a long half-life of the ALOX15 protein in human monocytes. To examine the importance of UTX mediated regulation of ALOX15 in human peripheral monocytes, UTX was knocked down by using lentivector shRNA methodology. Analysis of these cells showed that the IL-4-induced transcription of ALOX15 mRNA was significantly attenuated, indicating an important role of UTX in IL-4 induced ALOX15 expression also in human monocytes (Fig.4E upper panel). ChIP analysis was also carried out to verify the association of UTX with the ALOX15 promoter and PCR amplification of the DNA-protein binding region -500bp to -300bp revealed that IL-4 stimulation increases the binding of UTX to this promoter region. However, IL-4 stimulation did not change the H3K27me3 status at the ALOX15 promoter (Fig.? 4E lower panel), suggesting that UTX might also have an H3K27me3-demethylase-independent regulatory function in terms of ALOX15 induction in monocytes. Notably, a histone demethylase-independent function of UTX was recently described in mice [41], [42].

The HL cell line L1236 constitutively expresses ALOX15 at high levels and is a useful tool for the study of ALOX15 regulation and biologic function [19], [21], [31]. Interleukin-13 and the IL-13-specific receptor chain (IL-13R alpha1) are frequently expressed in HL-derived cell lines and in H-RS cells from biopsies of HL tissues. The activity of IL-13 is similar to that of IL-4 since the predominant receptor chain (IL-4R alpha) used is common to these two cytokines; the heterodimer of IL-4R alpha with IL-13R alpha1 is designated both as an IL-13 receptor and an IL-4 receptor [43]. Thus, the high expression level of ALOX15 in L1236 cells is in part thought to be a consequence of the autocrine pathway mediated by IL-13. Since UTX was shown to be an important mediator of IL-4-induced ALOX15 expression in both A549 cells and human peripheral monocytes, its potential role in the ALOX15 regulation in L1236 cells was investigated. UTX was detected in L1236 cells (data not shown) and ChIP assay revealed an association of UTX at the ALOX15 promoter, and furthermore, UTX depletion significantly inhibited the expression of ALOX15 (Fig.4F upper panel). However, UTX knockdown did not markedly influence on the methylation level of H3K27me3 (Fig.4F lower panel). Therefore, these results indicate that UTX is a common regulator of ALOX15 transcription, but that its mode of action seems to exhibit cell type-specific differences.

In summary, these findings illuminate the mechanisms involved in ALOX15 epigenetic regulation mediated by IL-4/IL-13 stimulation and not only demonstrate the critical role of UTX through specific H3K27me3 demethylation in A549 cells but also suggest UTX H3K27me3-demethylase-independent modulation in human monocytes and L1236 cells. Further studies aiming at characterization of the mechanisms associated with UTX demethylase-independent activation of ALOX15 are warranted.

## Supporting Information

Figure S1 **IL-4-mediated ALOX15 induction in A549 cells requires STAT-6.** (A) STAT-6 specific siRNA was transfected 72 hours prior to 48 hours IL-4 treatment, and total RNA was purified, followed by qRT-PCR, GAPDH was used as a loading control and the relative expression levels were calculated as the values relative to those of non-specific siRNA treated samples; (B)STAT-6 protein levels were measured by western blot, β-Actin was used as a loading control; (C, D) ALOX15 and UTX mRNA levels were measured by qRT-PCR after STAT-6 depletion followed by IL-4 treatment. qRT-PCR data is shown as “fold induction” relative to that in control cells. Error bars represent standard error mean of three independent experiments. *p<0.05; ** p<0.01; *** p<0.001.
(TIF)
