# Correction: Interleukin-4-Mediated 15-Lipoxygenase-1 Trans-Activation Requires UTX Recruitment and H3K27me3 Demethylation at the Promoter in A549 Cells

**DOI:** 10.1371/journal.pone.0091499

**Published:** 2014-02-28

**Authors:** The PLOS ONE Staff

In panel C of Figure 3, the indication bars for siCtrl and siUTX are missing. Please see the correct Figure 3 here.

**Figure 3:**
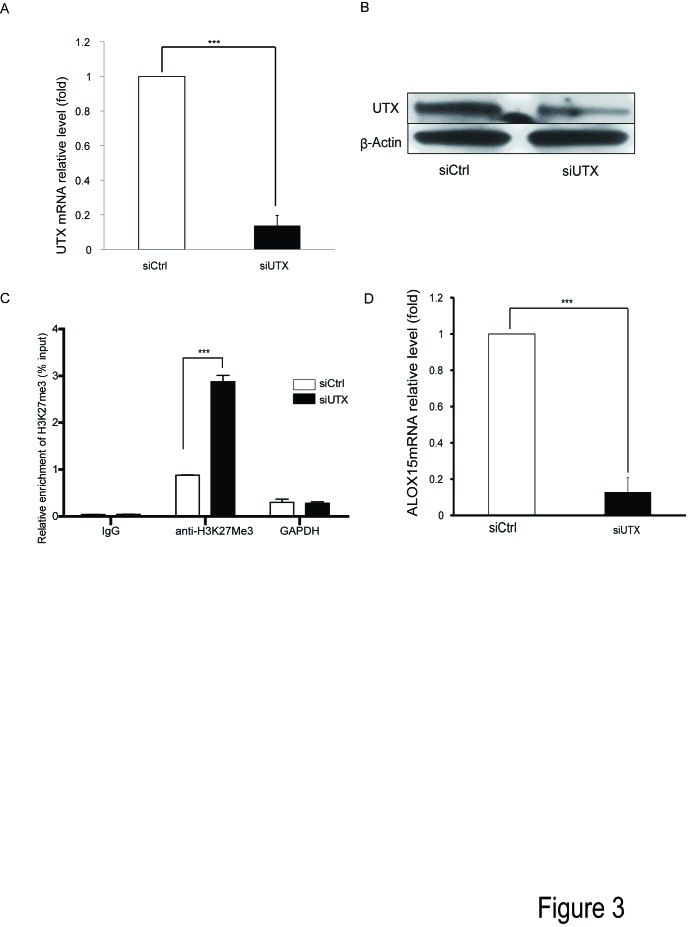
Lysine (K)-specific demethylase UTX is required for ALOX15 induction in A549 cells. UTX specific siRNA was transfected 72-4 treatment, and total RNA and protein were purified, followed by qRT-PCR and Western blot, the mRNA (A) and protein level (B) of UTX was measured upon UTX depletion followed by IL-4 stimulation. (C) The status of H3K27me3 at ALOX15 promoter region 3(see figure? 2) was verified upon UTX depletion followed by IL-4 stimulation; (D) the effect of UTX depletion on IL-4-induced ALOX15 expression was measured by qRT-PCR. All qRT-PCRs used GAPDH as loading control and the relative expression levels were calculated as the values relative to those of the calibrator samples (untreated sample). β-Actin was used as a loading control for all western blots. qRT-PCR data is shown as “fold induction” relative to that in control cells. Error bars represent standard error mean of three independent experiments. *p<0.05; ** p<0.01; *** p<0.001.

## References

[1] Han H, Xu D, Liu C, Claesson H-E, Björkholm M, et al (2014) Interleukin-4-Mediated 15-Lipoxygenase-1 Trans-Activation Requires UTX Recruitment and H3K27me3 Demethylation at the Promoter in A549 Cells. PLoS ONE 9(1): e85085 doi:10.1371/journal.pone.0085085 24465480 10.1371/journal.pone.0085085 PMC3896354

