# Supplementary figures and images for: Interleukin-4-Mediated 15-Lipoxygenase-1 Trans-Activation Requires UTX Recruitment and H3K27me3 Demethylation at the Promoter in A549 Cells
Source: PLoS One. 2014 Jan 20;9(1):e85085. doi: 10.1371/journal.pone.0085085 (PMC3896354; doi:10.1371/journal.pone.0085085)

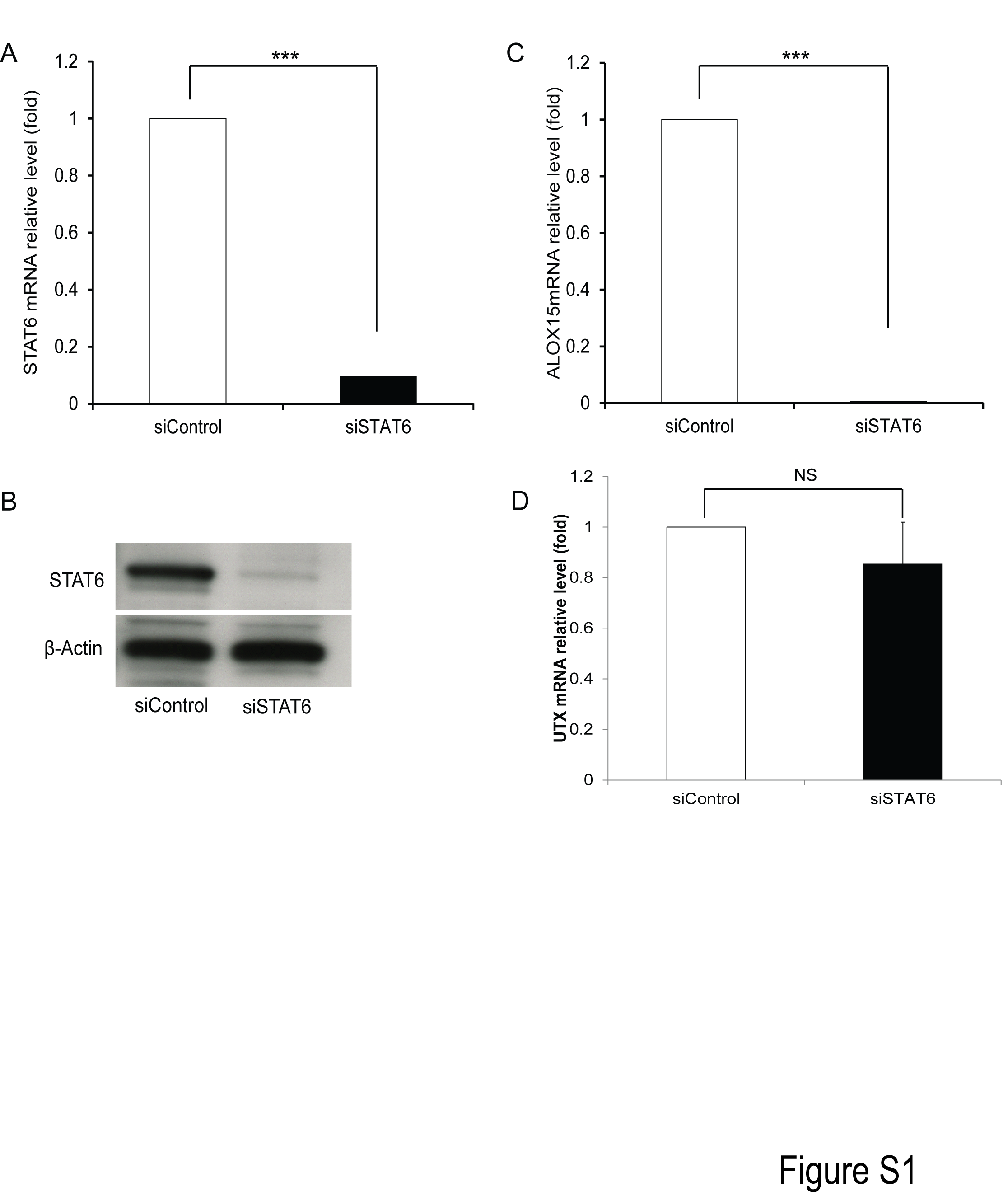

Supplement: Figure S1 — IL-4-mediated ALOX15 induction in A549 cells requires STAT-6. (A) STAT-6 specific siRNA was transfected 72 hours prior to 48 hours IL-4 treatment, and total RNA was purified, followed by qRT-PCR, GAPDH was used as a loading control and the relative expression levels were calculated as the values relative to those of non-specific siRNA treated samples; (B)STAT-6 protein levels were measured by western blot, β-Actin was used as a loading control; (C, D) ALOX15 and UTX mRNA levels were measured by qRT-PCR after STAT-6 depletion followed by IL-4 treatment. qRT-PCR data is shown as “fold induction” relative to that in control cells. Error bars represent standard error mean of three independent experiments. *p<0.05; ** p<0.01; *** p<0.001. (TIF) [file pone.0085085.s001.tif]
